# Supplementary material for: Magnitude and predictors of khat use among patients with tuberculosis in Southwest Ethiopia: A longitudinal study
Source: PLoS One. 2020 Jul 30;15(7):e0236154. doi: 10.1371/journal.pone.0236154 (PMC7392297; doi:10.1371/journal.pone.0236154)
Supplement: Attachment — Submitted filename: Factors associated with khat use among patients with tuberculosis in Southwest Ethiopia_Reviewer2_comments.docx [file pone.0236154.s001.docx]

**Study title: Factors associated with khat use among patients with tuberculosis in Southwest Ethiopia: A longitudinal study**

**General comment:**

The paper is generally well-written and adds to the broader literature on substance use and TB in Africa.  The findings may help readers better understand common factors that contribute to Khat use in this sub group; these insights may contribute to interventions. Although the paper may address a gap, there are several major weaknesses and the paper could be improved with a stronger background section, further elaboration of the methods, and a stronger and more well-organized discussion of the results. Specific comments follow:

**Title & Introduction**

The title is overly promising and yet the study mainly focuses on TB outpatients on DOTs. The authors should clarify the title further.

The burden of TB is fairly well described but the introduction is scanty of known risk factors for TB and khat use separately. In the discussion section, it might be worth highlighting if these risk factors are ant different from those found in this study, and why? This should be addressed.

**Methods**

Why was use of other illicit drug used a s an exclusion criteria?

The sources of selection of participants are clear but the and methods of both participant selection and follow up remain vague.

The authors need to clearly define khat use and to indicate sources of data and details of methods of assessment (measurement) and the rationale for the choice assessment. Indicate elsewhere in the paper that you refer to self-reported khat use not simply Khat use.

Your paper has many sources of selection bias from how participants were selected to how assessments of outcomes and exposures were done. Describe any efforts to address potential sources of bias.

Explain how the sample size was arrived at and the rationale underlying the models that were built during the analysis.

It remains unclear to me what conceptual framework underpinned the analysis, which confounders were known from before and how these were controlled for and why they were included.

**Results**

Would appreciate a note of missing data and how this was handled. At a minimum, Indicate the number of participants with missing data for each variable of interest

You need to show how the associations changed overtime-from baseline to endline and give plausible reasons.

**Discussion**

‘To our knowledge, this study is the first longitudinal study conducted among patients with tuberculosis investigating predictors of khat use in Africa’. Vague statement consider rephrasing.

Authors should discuss limitations of the study further, taking into account sources of potential bias or imprecision. Discuss both direction and magnitude of any potential bias.

**Conclusion:** ‘The findings imply that male patients and patients with alcohol use disorders should be focused on while screening for khat use among TB patients.’ I would argue that the goals of screening for khat use need to be much broader than male gender and AUD. Consider re phrasing this seemingly misleading statement.
