# Supplementary material for: Magnitude and predictors of khat use among patients with tuberculosis in Southwest Ethiopia: A longitudinal study
Source: PLoS One. 2020 Jul 30;15(7):e0236154. doi: 10.1371/journal.pone.0236154 (PMC7392297; doi:10.1371/journal.pone.0236154)
Supplement: Attachment — Submitted filename: Review comments.docx [file pone.0236154.s002.docx]

Thank you for giving the opportunity to review this manuscript titled “Factors associated with khat use among patients with tuberculosis in Southwest Ethiopia: A longitudinal study” [PONE-D-20-01650]

Dear authors, Good study surely based on the needs of your area! Please consider the followings

1- Overall, this is an interesting study exploring the use of plants (khat) for stimulation purpose among patients with tuberculosis. However, it is unclear whether the use among the local population is mainly recreational, or due to its medicinal properties. Does Khat chewing has any scientific explanation to causes addiction?
2- Dear authors, I am great full for your efforts. But, your main massage is to demonstrate khat use by focusing on epidemiological data and factors associated with it among tuberculosis patients. However, as it is explained in your background information, chewing khat is not uncommon including tuberculosis patients. If so, I believe that the big deal is not reporting the magnitude or level of khat use, rather, since your study is longitudinal, the better was to give important intervention and to see its effect. This might helpful to recommend for policy makers and other stakeholders at national level. –

otherwise, you should write more about the gap in your context and the real problem which motives you to do such a study. Problem statement is not convincing! The clear gap to see the prevalence of khat use and factors affecting it at different stages is not well explained.

3- say the evidences that emphasize on the importance of to take preventive measures and reduces medical complications related tuberculosis that might be caused by Khat consumption during medication follow-up apart from non-adherence (found in your study)?
4- In your study, there is nothing intervention or education is given for participants i.e. not indicated in your writing, however, varied findings are documented regarding the prevalence of khat use at baseline and last data collection time. What sort of things can be reasons to decrease the prevalence (in percentile)? And writ more about this
5- This study reported that the use of Khat among patients with tuberculosis & alcohol problem is higher. Please discuss further whether this something good or bad, because it may divert the individuals from using other more harmful substances.
6- In your exclusion criteria………polysubstance users (using two or more substances) and patients were excluded from the study. However, alcohol use problem (disorder) is a predictor to use khat. It is unclear and ambiguous idea. Could you say more about this please? (Since the participants in your study were using both khat and alcohol)
7- Also in your sampling procedure regarding to exclusion and inclusion criteria, it is stated as…..We included all patients who had recently been diagnosed with TB who had started DOT in the selected health centers and hospitals. Patients were included only if they had started TB treatment within less than four weeks before inclusion and were not planning to transfer to other health institutions. Patients aged less than 18 years, patients infected with multidrug-resistant TB strains ,………..were excluded from the study. Reason behind to include recently diagnosed (4 weeks) only?? Does it have any significance difference to chew khat with people living with TB for long?????
8- In your data collection procedure part, It is first time Agaro is mentioned. No context provided as to why pre-testing was done in Agaro (which reader outside Ethiopia may not know why it is important for a study). Write more about Agaro where it is located??
9- Doe the pretest was done using both version of the questionnaires (Afan oromo and Amharic). ???
10- Self-Reporting Questionnaire (SRQ-20) to assess mental distress, -WHO's structured questionnaire of Alcohol Use Disorder Identification Test (AUDIT) to asses Alcohol Use Disorders and questions used to assess the frequency and patterns of Khat use- Operational definitions should be about measures items, dimensions, rating scales, theoretical basis, validity, reliability and so on...

I thought it seems to write it again deeply to increase the quality.

11- Prevalence of khat use at baseline and last assessment examined different (39.2% Vs 37.3%). What it its implication?? Clearly not explained specifically among tuberculosis patients? I suggest to discuss it more including thee pattern of khat chewing.
12- To show factors associated with khat chewing I suggest to include confidence interval also so that it will be clear more.
13- Try to discuss your findings detail……….. About merchants (why they chew more….. the prevalence of khat use if there is study findings done longitudinally…..)??? discussion is limited.
14- Lastly …I thought it looks scientifically sound. If your title re written as “Magnitude and factors associated with khat use among patients with tuberculosis in Southwest Ethiopia: A longitudinal study”
