# Supplementary material for: Magnitude and predictors of khat use among patients with tuberculosis in Southwest Ethiopia: A longitudinal study
Source: PLoS One. 2020 Jul 30;15(7):e0236154. doi: 10.1371/journal.pone.0236154 (PMC7392297; doi:10.1371/journal.pone.0236154)
Supplement: Attachment — Submitted filename: Response to Reviewers-Plos_Khat.docx [file pone.0236154.s004.docx]

**Response to reviewers**

**Reviewer #1**

This paper tries to explore the factors associated with Khat use among tuberculosis patients. However the paper has serious flaws of research design and rationale. It lacks coherency in terms of objectives of research. What factors authors are trying to associate??

Following are the specific comments.

1. The stated objective of the research is “there is limited information on factors associated with Khat use among patients with TB and this study aimed at addressing this research gap.” But data and conclusion doesn’t support it

**Response**: Indeed we wanted to examine predictors of Khat use longitudinally, and we apologize if this was not completely clear. Our results showed that being male, being a government employee and the presence of alcohol use disorders were associated with khat use among TB patients. To our knowledge, this is one of the few studies that investigated a broad range of potential predictors. This can be found on pages 12 and 13 line 1 to 8.

2. Study design: The study design doesn’t support the curation of factors associated with Khat use.

**Response**: Data originates from a comprehensive cohort study, so the longitudinal aspect was incorporated. Cohort studies are generally state-of-the-art for investigating risk factors.

3. Sample size: the sample size is not adequate enough to draw such broad conclusion.

**Response**: This is a valuable comment. We now mention this as a limitation of the study which is found on page 18, lines 9 to 13 before the topic “conclusions”. The sample size was calculated based on previous studies with a prevalence of almost 50%. We found a prevalence of about 40%, an acceptable difference. For regression models, 10 cases (here: patients using khat) per predictor to be included is generally sufficient. Thus, our sample size allows models with up to 10 predictors which are well in line with our specifications.

The title and the subsequent research methodology adopted do not match at all.

**Response**: We have amended the title as “ magnitude and predictors of khat use among patients with tuberculosis in Southwest Ethiopia: A longitudinal study” and this found the first page of the manuscript.

4. There are language issues but they are of minor nature.

**Response**: We have found a few errors and edited them throughout the manuscript.

**Reviewer #2**

General comment:

The paper is generally well-written and adds to the broader literature on substance use and TB in Africa. The findings may help readers better understand common factors that contribute to Khat use in this sub group; these insights may contribute to interventions. Although the paper may address a gap, there are several major weaknesses and the paper could be improved with a stronger background section, further elaboration of the methods, and a stronger and more well-organized discussion of the results.

Specific comments follow:

Title & Introduction
1. The title is overly promising and yet the study mainly focuses on TB outpatients on DOTs. The authors should clarify the title further.

**Response**: We agree and have amended the title as “Magnitude and predictors of khat use among patients with tuberculosis in Southwest Ethiopia: A longitudinal study”. The amendment regarding the topic is found on the **first page** of the manuscript. However, the reason why we have included only DOTs patients is that there is no chance that patients who are getting treatment at the inpatient department would access substance including khat. Also, we have already included this issue in limitation of the study stating that the findings of this study will not be generalized to those patients who are getting treatment at the inpatient department.

2. The burden of TB is fairly well described but the introduction is scanty of known risk factors for TB and khat use separately. In the discussion section, it might be worth highlighting if these risk factors are ant different from those found in this study, and why? This should be addressed.

**Response**: We have accepted the reviewer’s comment and amended the introduction but there was no difference from what was found in the past in terms of risk factors except beliving that khat could reduce medication sided effects and TB related symptoms and this is already available in the discussion. We have added risk factors that affect khat use to the introduction and discuss that are included in our study and associated with khat use. The amendment to the introduction found in the second paragraph page 4 to 5 line 5 to 9.

##Methods

3. Why was use of other illicit drug used a s an exclusion criteria?

**Response:** We did not exclude the use of illicit drugs but unfortunately we could not find a patient who uses illicit drug and this could be related to availability or fear of legal consequences.

4. The sources of selection of participants are clear but the and methods of both participant selection and follow up remain vague.

**Response**: Thank you for your comments. The participants who fulfill the inclusion criteria were invited to participate in the study consecutively and Interviews were conducted within the respective health institutions when the participants came for their TB clinic visits. The follow up was done at the end of two and six months. The changes are made under the topic of “**Data collection procedure”** lines 7 to 10 and 13 to 14 on page 7.

5. The authors need to clearly define khat use and to indicate sources of data and details of methods of assessment (measurement) and the rationale for the choice assessment. Indicate elsewhere in the paper that you refer to self-reported khat use not simply Khat use.

**Response**: We have accepted the comment and amend the manuscript. In our study khat use was defined as using khat in the past 30 days and we have included a reference for this. We have also indicated the source of data and sample size calculations in the manuscript. Likewise, the rationale for the choice assessment added to the manuscript. Throughout the manuscript, we have used the term khat use. The changes regarding the definition of khat use are available under the topic “**Data collection tools”** on page 8 line 2 to 4. Also, the changes regarding the source of data and detail of the method of assessment are found under the topic “**Study population and sampling procedure”** on page 6 lines 4 to 5, and on pages 6 and 7, lines 8 to 17.

6. Your paper has many sources of selection bias from how participants were selected to how assessments of outcomes and exposures were done. Describe any efforts to address potential sources of bias.

**Response**: We are grateful for the reviewer's comment. Since we have selected the participants consecutively participants who have fulfilled the inclusion criteria had an equal chance to be involved in the study. So, there was a limited selection and assessment of outcomes and exposures bias.

7.Explain how the sample size was arrived at and the rationale underlying the models that were built during the analysis.

**Response**: We have accepted the comment and amended the manuscript. The sample data for this study was drawn from a cohort registered as ጤ/ኢ/ም/ድ/ም/ዳ/476/2011 which aimed to assess substance use disorder, quality of life, mental health and adherence to anti-TB medication. The sample was calculated considering power=80%, 95% confidence interval, 62.4% prevalence of non-adherence to anti-TB medications among khat users, 43.6% prevalence of non-adherence among non-khat user TB patients, and 20% of drop out. The total sample size was 268 where 105 use khat and 163 were not. Also, the model was built considering theoretical importance and the adequate number of participants in each cell for each category. So, we have included this issue in the manuscript. The changes are found under the topic “**Study population and sampling procedure**” on page 6 and 7 lines 11to 15 and under the topic “**Data analysis**” on page 9 and 10, line 3 to 5

8.It remains unclear to me what conceptual framework underpinned the analysis, which confounders were known from before and how these were controlled for and why they were included.

**Response**: The variables with potential confounders were controlled using multivariable modeling in which we have included potential variables that could affect khat use. We used risk factors that were known from the literature. Therefore, the findings have been adjusted for potential confounders. For example, gender and age and others which are found in table 2 page 14. The model was built based on the theoretical importance and the adequate number of participants in each cell for each category. The changes to the analysis are found on pages 9 and 10, lines 2 to 5.

\##**Results**

9.Would appreciate a note of missing data and how this was handled. At a minimum, indicate the number of participants with missing data for each variable of interest

**Response**: We have used the case-wise deletion of participants with missing data. Hence, 22 participants have been omitted from the analysis because of missing data on one or more variables. We have amended the manuscript accordingly and the changes are found under the “**Analysis**” on page 10, line 4 o the analysis and under the “result” on page 11, line 7

10. You need to show how the associations changed overtime-from baseline to endline and give plausible reasons.

**Response**: As it is indicated in table 2 the strength of the association was improved over time as witnessed by further improvement of the model fit (BIC=815.7). The changes were made on pages 12 line 2 to 3.

**##Discussion**

11.‘To our knowledge, this study is the first longitudinal study conducted among patients with tuberculosis investigating predictors of khat use in Africa’. Vague statement consider rephrasing.

**Response**: We have accepted the comment and edit it as follows: “Our longitudinal study is first in Africa to investigate predictors of khat use among patients with TB.” The changes are found in the first line of the discussion on page 15.

12. Authors should discuss limitations of the study further, taking into account sources of potential bias or imprecision. Discuss both direction and magnitude of any potential bias.

**Response**: We are grateful for the reviewer's comment and added some additional potential limitations. Also, we want to inform the reviewer as we have reported the following bias: social desirability bias, data collection by health professionals working in the clinic, tools used to assess reported khat use, etc. The changes are available on page 18 line 9 to13.

**##Conclusion**

13. ‘The findings imply that male patients and patients with alcohol use disorders should be focused on while screening for khat use among TB patients.’ I would argue that the goals of screening for khat use need to be much broader than male gender and AUD. Consider re phrasing this seemingly misleading statement.

**Response**: We agree with the reviewer's comment that the screening of patients for khat use has to be comprehensive, but our conclusion is only based on the findings of the study. We rephrased the sentence with caution. The conclusion now reads as ”The findings indicate screening for khat use among TB patients needs to prioritize males and those with alcohol use disorders.” The changes are made under the topic of “**Conclusions**” on page 18 line 18 to 20 and 22 to 23.

**Reviewer #3**

Thank you for giving the opportunity to review this manuscript titled “Factors associated with khat use among patients with tuberculosis in Southwest Ethiopia: A longitudinal study” [PONE-D-20-01650]

Dear authors, Good study surely based on the needs of your area! Please consider the followings

1. Overall, this is an interesting study exploring the use of plants (khat) for stimulation purpose among patients with tuberculosis. However, it is unclear whether the use among the local population is mainly recreational, or due to its medicinal properties. Does Khat chewing has any scientific explanation to causes addiction?

**Response**: Thank you for the constructive comment. Khat can cause psychological dependence among long time users and we have included this in the introduction. Also, local people use khat for recreational, social lubrication, to reduce the feeling of hunger, as self-medication, and to stay alert while praying. We have already included some of these factors while we were discussing our results. The changes are found on page 4 second paragraph line 2 to 5 and page 5 line 5 to 10.

2. Dear authors, I am great full for your efforts. But, your main massage is to demonstrate khat use by focusing on epidemiological data and factors associated with it among tuberculosis patients. However, as it is explained in your background information, chewing khat is not uncommon including tuberculosis patients. If so, I believe that the big deal is not reporting the magnitude or level of khat use, rather, since your study is longitudinal, the better was to give important intervention and to see its effect. This might helpful to recommend for policy makers and other stakeholders at national level. –otherwise, you should write more about the gap in your context and the real problem which motives you to do such a study. Problem statement is not convincing! The clear gap to see the prevalence of khat use and factors affecting it at different stages is not well explained.

**Response**: We have accepted about explaining the statement of the problem further and amended the manuscript. But, arguably, there are not many studies targeting khat use among patients with tuberculosis. There is also a scarcity of information regarding the magnitude of khat use from longitudinal studies as many of the studies are cross-sectional. Also, those studies did not assess khat use primarily considering it as the main outcome variable but they report khat use on their way to assess other problems among TB patients. Our data provide evidence for continued use of khat among TB outpatients despite regular contact with health professionals so that we make the case for more structured and effective interventions to be developed and tested. Regrettably, our study was not designed as an intervention study, but the reviewer is correct to state that this should be the aim of further research, and this study is most likely to inform future trials in this regard. The changes we have made are available in the second paragraph of the introduction on page 5 line 8 and in the last 8 sentences of the introduction.

3. Say the evidences that emphasize on the importance of to take preventive measures and reduces medical complications related tuberculosis that might be caused by Khat consumption during medication follow-up apart from non-adherence (found in your study)?

**Response**: We have studied the effect of khat use among tuberculosis including non-adherence but we have prepared a separate manuscript which gives detail of this information.

4. In your study, there is nothing intervention or education is given for participants i.e. not indicated in your writing, however, varied findings are documented regarding the prevalence of khat use at baseline and last data collection time. What sort of things can be reasons to decrease the prevalence (in percentile)? And writ more about this

**Response**: We have accepted the comment partly and amend the manuscript accordingly. Our study is not an interventional study so we did not primarily focus on intervention, but we have trained our data collectors to give education regarding problematic khat use and its effect at the end of data collection. During data collection, we have advised those who use khat more frequently to talk to a mental health professional and we have included this under the topic of “ethical consideration”. We added the probable reason why khat use was declined over time to the discussion part. The amendment is found on page 10 in the last sentence of ethical consideration and in the discussion, on page15, the second paragraph line 8 to 13.

5. This study reported that the use of Khat among patients with tuberculosis & alcohol problem is higher. Please discuss further whether this something good or bad, because it may divert the individuals from using other more harmful substances.

**Response**: We have accepted the comment and made discussion as follows: Combining alcohol and khat would affect patients' treatment outcomes, and lead to physical and mental health problems. Because both khat and alcohol were found to have a potential impact on the immunity of the user and associated with mental distress so that patients may develop severe medical and mental health complications or die earlier than non-users. Furthermore, these two substances have an association with treatment-resistant tuberculosis. The amendment is found on page 17, lines 2 to 7.

6. In your exclusion criteria………polysubstance users (using two or more substances) and patients were excluded from the study. However, alcohol use problem (disorder) is a predictor to use khat. It is unclear and ambiguous idea. Could you say more about this please? (Since the participants in your study were using both khat and alcohol)

**Response**: We are grateful for the comment. We have excluded patients who were using three or more substances (polysubstance users) and amended the manuscript accordingly. We are sorry for the typing error. The amendment is found under the topic of “**Study population and sampling procedure”** page 6, line 7 and now read as “using three or more substances”

7. Also in your sampling procedure regarding to exclusion and inclusion criteria, it is stated as…..We included all patients who had recently been diagnosed with TB who had started DOT in the selected health centers and hospitals. Patients were included only if they had started TB treatment within less than four weeks before inclusion and were not planning to transfer to other health institutions. Patients aged less than 18 years, patients infected with multidrug-resistant TB strains ,………..were excluded from the study. Reason behind to include recently diagnosed (4 weeks) only?? Does it have any significance difference to chew khat with people living with TB for long?????

**Response**: Since we were also following other outcomes such as adherence, mental health, and quality of life, we have decided to include only new patients because it allows us to see any change over time. Also, the phase of treatment is important because in the first few weeks since patients have serious symptoms of tuberculosis we want to see whether patients increase or decrease their khat consumption. Furthermore, it is difficult to include other patients such as those who are on retreatment and MDR-TB because the duration of treatment is quite different and we have already included this in the limitation of the study.

8. In your data collection procedure part, It is first time Agaro is mentioned. No context provided as to why pre-testing was done in Agaro (which reader outside Ethiopia may not know why it is important for a study). Write more about Agaro where it is located??

**Response**: We have accepted the comment and now reads as ….Agaro (a town found in Jimma zone at a distance of about 45 kilometers from Jimma city). The changes are found under the topic of “**Data collection procedure”** on page 7, line 3.

9. Doe the pretest was done using both version of the questionnaires (Afan oromo and Amharic). ???

**Response**: Yes we have done the pretest and data collection in both languages because our participants speak either of the two languages. The changes are found under the topic of “**Data collection procedure**” on page 7, line 14 (last sentence).

10. Self-Reporting Questionnaire (SRQ-20) to assess mental distress, -WHO's structured questionnaire of Alcohol Use Disorder Identification Test (AUDIT) to asses Alcohol Use Disorders and questions used to assess the frequency and patterns of Khat use- Operational definitions should be about measures items, dimensions, rating scales, theoretical basis, validity, reliability and so on...
    I thought it seems to write it again deeply to increase the quality.

**Response**: We have accepted the comment and added detail information about the tools such as validity, specificity, sensitivity, and cut-off points into the method section of the manuscript. Also, we have included supporting references. The changes are found under the topic of “**Data collection tools**” on pages 8 to 9 in the third paragraph line 2 to 8 and on page 9, paragraph 3 lines 2 to 9.

11. Prevalence of khat use at baseline and last assessment examined different (39.2% Vs 37.3%). What it its implication?? Clearly not explained specifically among tuberculosis patients? I suggest to discuss it more including thee pattern of khat chewing.

**Response**: We have accepted the comment and amended the manuscript. The finding indicates the decline of khat use over time which is not significant enough. The pattern of khat use could change over time because patients might use more khat at baseline as a self-treatment for tuberculosis related symptoms and medication side effects. Because we have identified most patients believe khat can reduce symptoms of tuberculosis and medication side- effects. So, when they get improvement from the disease they might reduce their khat use. The changes are found in the discussion page 15 paragraph 2, lines 8 to 13.

12. To show factors associated with khat chewing I suggest to include confidence interval also so that it will be clear more.

**Response**: We have accepted the comment and included confidence intervals in Table 2 and it is found on page 14.

13. Try to discuss your findings detail……….. About merchants (why they chew more….. the prevalence of khat use if there is study findings done longitudinally…..)??? discussion is limited.
    **Response**: We have accepted the comment and added the explanation of why merchant chews khat more. So, in the discussion, we have included the following points: Merchants use khat to be alert and energetic at the workplace. Also, some of the merchants may be khat sellers, and, as a result, they chew khat to attract customers but further study assessing this situation is needed. The changes are found on page 17 in the third paragraph line 15 to 19.
14. Lastly …I thought it looks scientifically sound. If your title re written as “Magnitude and factors associated with khat use among patients with tuberculosis in Southwest Ethiopia: A longitudinal study”

**Response**: We have accepted the comment and amended the title as “Magnitude and predictors of khat use among patients with tuberculosis in Southwest Ethiopia: A longitudinal study” The change is found on the first page of the manuscript.
