# Supplementary material for: Magnitude and predictors of khat use among patients with tuberculosis in Southwest Ethiopia: A longitudinal study
Source: PLoS One. 2020 Jul 30;15(7):e0236154. doi: 10.1371/journal.pone.0236154 (PMC7392297; doi:10.1371/journal.pone.0236154)
Supplement: Attachment — Submitted filename: V5. Response Reviewers.docx [file pone.0236154.s005.docx]

**Language:** This manuscript is edited by a professional English language editor.

**Response to reviewer**

**Comment [A1]:** Could shorten title further:

“Magnitude and predictors of khat use among tuberculosis patients in Southwest Ethiopia: A longitudinal study”

**Response:** Thank you for the comment. We would like to keep the current title because we are concerned that further shortening of the title may make it less informative about the objectives, the area and design of the study.

Comment [A2]: Limited chance perhaps?

**Response**: We agree with the reviewer's comment because there is limited chance for TB inpatients access to substances including khat. We have included this issue in the discussion section on page 18, line 12 to 14 .

Comment [A3]: This change doesn’t show in the limitations section. Please revise.

**Response**: Thank you for your comment. This information was stated on page 18, lines 4 to 7 on the previous version of the manuscript. Now we have separately reported it on page 18, line 9 to 10 as “The findings of this study can not be generalized to those patients who are getting treatment at the inpatient facilities”

Comment [A4]: This response is unsatisfactory.

**Response**: On the top of theoretical importance and the adequate number of participants in each cell for each category, we have also used literature, clinical knowledge, and experience to build the model. The reason why we have included confounders is that they have the potential to distort the association between dependent and independent variables. Also, it is difficult to identify variables that independently predict khat use without controlling for confounders. Therefore, we have controlled them using multivariable analysis. We have made additional clarification to the analysis on page 9 lines 3 to 4.

Comment [A5]: Please show this missing data as a footnote to the table

**Response**: we have accepted and made changes on page 11.

Comment [A6]: Consider: “To our knowledge, this study is the first longitudinal study investigating predictors of khat use conducted among patients with tuberculosis in Africa.”

**Response**: We have accepted the comment and made changes on page 15, lines 1 and 2.

Comment [A7]: The author does not address issues of the direction of potential biases listed. This is a major omission in my view.

**Response:** We have reported the direction of bias on 18, lines 2 to 4 and now read as “Due to social desirability, patients may minimize or deny their khat consumption, which could have a negative impact on the extent of khat use. It could also affect the association of predictors with the outcome variables. In addition, health professionals working in a TB clinic might have contribute to this bias, as patients may be inclined not to report their khat use or to minimize its amount.

Comment [A8]: From your findings, it seems that screening for Khat is a major conclusion itself. Paying attention wo men and people with AUD is secondary right?

**Response:** We have rephrased the conclusion as “All patients diagnosed with TB should be screened for khat use and a particular emphasis should be given to males and individuals with a history of alcohol use” The changes are available on page 2, lines 2 and 3 and on page 18, lines 4 and 5.”
